# Collagen Peptides and *Saccharomyces boulardii* CNCM I‐745 Attenuate Acetic Acid‐Induced Colitis in Rats by Modulating Inflammation and Barrier Permeability

**DOI:** 10.1002/fsn3.70189

**Published:** 2025-04-18

**Authors:** Öykü Altınok, Murat Baş, Elif Gelenli Dolanbay, Meltem Kolgazi, Tugay Mert, Ünal Uslu

**Affiliations:** ^1^ Department of Nutrition and Dietetics Institute of Health Sciences, Acibadem Mehmet Ali Aydinlar University Istanbul Turkey; ^2^ Department of Nutrition and Dietetics, Faculty of Health Sciences Fenerbahçe University Istanbul Turkey; ^3^ Department of Nutrition and Dietetics, Faculty of Health Sciences Acibadem Mehmet Ali Aydinlar University Istanbul Turkey; ^4^ Department of Histology & Embryology, School of Medicine Istanbul Medeniyet University Istanbul Turkey; ^5^ Department of Physiology, School of Medicine Acibadem Mehmet Ali Aydinlar University Istanbul Turkey

**Keywords:** collagen peptides, inflammation, rat, *Saccharomyces boulardii*, ulcerative colitis

## Abstract

Ulcerative colitis (UC) is an inflammatory bowel disease characterized by recurrent episodes of inflammation and tissue damage, with limited treatment options. This study aimed to investigate the effects of collagen peptides and *Saccharomyces boulardii* on acetic acid (AA)‐induced colitis. Thirty‐six male Sprague–Dawley rats were randomly divided into the following four groups: normal control (NC), colitis control (CC), collagen peptide (CP; 0.6 g/kg/day), and *S. boulardii* (SB; 250 mg/day). Colitis was induced by an intrarectal administration of AA in all groups except NC, and treatments were administered daily for 7 days. The therapeutic effects were evaluated by assessing the disease activity index (DAI), colon mass index, macroscopic and microscopic tissue damage, histopathological changes, zonula occludens (ZO)‐1 protein expression, and myeloperoxidase (MPO) activity. The results showed that CP and SB treatments substantially alleviated DAI scores (*p <* 0.05) and reduced the colon mass index. Colon macroscopic and microscopic damages improved compared to the CC group (*p <* 0.01). Histologically, both treatments reduced inflammatory cell infiltration, crypt damage, and ulceration, with CP showing a slightly more pronounced effect. Immunohistochemical analysis revealed significant restoration of ZO‐1 protein expression in the treated groups, indicating improvement in intestinal barrier integrity (*p <* 0.01). Furthermore, MPO activity was reduced in both CP and SB groups, significantly in the SB group (*p <* 0.01). These findings are consistent with previous studies that highlight the anti‐inflammatory and barrier‐enhancing effects of collagen peptides and probiotics in UC models.

## Graphical abstract

Collagen peptides and *Saccharomyces boulardii* CNCM I‐745 reduced colon damage and significantly alleviated acetic acid‐induced colitis in rats by restoring ZO‐1 expression and suppressing MPO activity. Collagen peptides and *S. boulardii* are potential therapeutic candidates for the treatment of colitis.

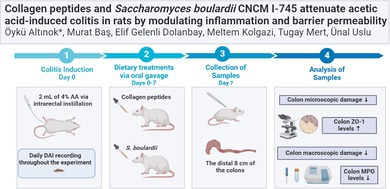

## 1 Introduction

Ulcerative colitis (UC) is a type of inflammatory bowel disease (IBD) characterized by progressive and widespread inflammation of the colon, leading to pathological mucosal damage and ulceration (Kobayashi et al. 2020). The most common clinical manifestations of UC include weight loss, bloody diarrhea, abdominal pain, rectal bleeding, tenesmus, and fatigue (Segal et al. 2021). If left untreated or inadequately managed, UC can progress to colorectal cancer (Trivedi et al. 2018). Diagnosis relies on a combination of clinical, biological, endoscopic, and histological findings (Asaad and Mostafa 2024). The prevalence of UC varies geographically (Ooi et al. 2019), and it is a leading cause of morbidity, particularly in industrialized countries (Ng et al. 2017). In recent years, its incidence has risen sharply in Asian countries, presenting a significant global health challenge (Ooi et al. 2019).

The pathophysiology and etiology of UC remain complex and not fully understood (Wallace et al. 2014). In genetically predisposed individuals, environmental factors such as lifestyle contribute to intestinal microbiota dysbiosis and increased colon epithelial permeability, triggering an abnormal immune response (Hassan et al. 2024). This response leads to neutrophil infiltration and the release of cytokines and other mediators, which cause colon tissue damage and contribute to disease progression. Overall, oxidative stress and inflammation play critical roles in UC pathogenesis (Merga et al. 2014).

The primary therapeutic goal in UC treatment is to establish a rapid clinical response and normalize biomarkers, followed by achieving endoscopic recovery to maintain clinical remission for a long time (Kobayashi et al. 2020; Asaad and Mostafa 2024). The choice of medications is based on disease extent and severity, response to previous or current therapy, and the presence of complications (Ardizzone et al. 2010). Four major drug classes are currently used to manage UC: aminosalicylates, immunosuppressants, glucocorticoids, and biologic agents (Liu et al. 2022). However, despite available pharmacological treatments, no definitive cure has been identified, and none of the existing drugs can completely eradicate the disease (Samuel et al. 2013). Instead, these medications help induce and maintain remission, reduce the risk of complications, and improve patients' quality of life during their administration (Gajendran et al. 2019). However, they present several limitations, including the development of drug resistance with long‐term use, significant side effects, treatment failure, and high economic costs (Oka and Sartor 2020). Consequently, ongoing research aims to develop therapeutic strategies with fewer adverse effects and improved clinical outcomes (Fakhoury et al. 2014).

Recently, researchers have increasingly focused on dietary agents that modulate inflammation and oxidative stress as potential adjuncts in UC treatment. Several dietary components have demonstrated benefits, including omega‐3 fatty acids (Marton et al. 2019), curcumin (Masoodi et al. 2018), amino acids (Liu et al. 2017), probiotics (Guo et al. 2024), and collagen peptides (Xing et al. 2022). Although these dietary agents show promise in UC management, current evidence remains insufficient to recommend their routine clinical use (Radziszewska et al. 2022).

Collagen protein is the most abundant structural protein in the extracellular matrix of the intestinal mucosa. Collagen peptides are low‐molecular weight peptides formed by the enzymatic hydrolysis of collagen (Gelse et al. 2003). They contain more glycine, glutamic acid, proline, and arginine (Chen, Hou, et al. 2017; Chen, Zhou, et al. 2017). Collagen peptides have been shown in studies on various colitis models to significantly reduce inflammation, improve colon barrier integrity, and help regulate colon microbiota composition (Rahabi et al. 2022; Xing et al. 2022). Vertebrates; however, have 28 collagen types composed of various polypeptide chains (Ricard‐Blum 2011). These collagen types have significantly different sizes, functions, and tissue distributions (Gelse et al. 2003). In mammals, Types I and III collagens account for 75%–90% of total collagen (Rahabi et al. 2022). The effects of collagen peptides differ in their source, dosage, and duration of supplementation. Further research is needed to understand the mechanisms of action of collagen peptides as potential therapeutics in UC (Li et al. 2022).

Certain probiotics have been shown in studies to be both protective and pathogenic in the development of UC (Guo et al. 2024). Probiotics are an excellent strategy for treating UC by regulating the colon microbiota and mucosal immune responses (Dhillon and Singh 2020). *Saccharomyces boulardii* CNCM I‐745 is a nonpathogenic probiotic yeast strain of *Saccharomyces cerevisiae* , classified as a facultative anaerobic fungus. Its activity in antibiotic‐associated infectious and functional diarrhea has been well documented (Terciolo et al. 2019). In rodent colitis models, it reduced disease severity, colon damage, and inflammation while promoting colon epithelial integrity (Rodríguez‐Nogales et al. 2018; Dong et al. 2019). Experimental studies have shown that it is a promising probiotic candidate for UC (Kelesidis and Pothoulakis 2012; Sivananthan and Petersen 2018). However, the precise mechanism of *S. boulardii* in colitis is not yet well understood and requires further investigation (Zhou et al. 2018; Dong et al. 2019).

To date, no studies have examined the effects of *S. boulardii* CNCM I‐745 and collagen peptides in acetic acid (AA)‐induced colitis. This study aimed to evaluate the therapeutic potential of dietary collagen peptides and *S. boulardii* CNCM I‐745 in a rat model of AA‐induced colitis. By investigating their efficacy, we seek to provide new insights into UC management and contribute to the development of more effective, low‐risk treatment strategies for this debilitating condition.

## 2 Materials and Methods

### 2.1 Experimental Animals

Sprague–Dawley rats (250–300 g; males) were used for the experiments and were supplied by the Acibadem Mehmet Ali Aydinlar University Laboratory Animal Research Center (Istanbul, Turkey). The rats were fed standard pellets and tap water *ad libitum* and kept in controlled conditions (12‐h light and dark cycles, temperature 22°C ± 2°C, and 65%–70% humidity). The experimental protocols involving animals received approval from the Yeditepe University Local Ethics Committee (2021/08‐10).

### 2.2 Experimental Design

**FIGURE 1:**
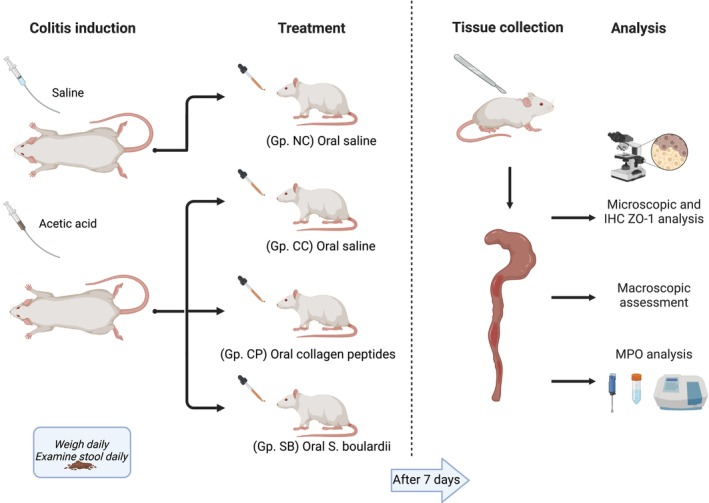
Timeline of colitis induction and treatments. NC, normal control; CC, colitis control (*n* = 9); CP, collagen peptide (*n* = 9); SB, *S. boulardii* (*n* = 9); MPO, myeloperoxidase; ZO‐1, zonula occludens‐1.

The rats were divided into the following four groups, each comprised of nine rats: normal control (NC), colitis control (CC), collagen peptide (CP), and *S. boulardii* (SB). Following 24 h fasting, colitis was induced with 2 mL of 4% AA via intrarectal instillation at 8 cm proximal to the anus under mild isoflurane anesthesia. To prevent leakage of the solution, the rats were kept in the Trendelenburg position for 30 s (Soliman et al. 2018). The NC group underwent the same procedure, but with saline instead of AA. Dietary treatments were given via oral gavage starting 2 h after intrarectal instillation and continued for 7 days. The CP and SB groups received 0.6 g/kg of bovine collagen peptide (Nature's Supreme, Türkiye) and 250 mg of *S. boulardii* (5 × 10^9^ CFU, Reflor, İstanbul), respectively, dissolved in 1 mL of saline. The NC and CC groups received 1 mL of saline daily. The rats were sacrificed by intense isoflurane inhalation at 24 h following the last gavage. The experimental protocol applied is shown in Figure 1.

After a midline incision, the distal 8 cm of the colon was excised 2 cm above the anal margin, opened longitudinally, and washed with saline. Colon samples were weighed and damage of tissues was scored macroscopically. Samples were divided into two equal parts transversely. The distal 4 cm portion of the samples was kept in 10% (v/v) formalin for histologic and immunohistochemistry (IHC) analysis, and the proximal 4 cm portion was stored at −80°C for biochemical analysis.

### 2.3 Disease Activity Index

Body weight, stool consistency, and blood in the stool were recorded daily. The difference between the final and initial weights of the rats was calculated. Each rat was scored as follows: 0, no weight loss, well‐formed stool, and/or no bloody stool; 1, 1%–5% weight loss; 2, 5%–10% weight loss, semiformed stool, and/or occult blood in the stool; 3, 10%–15% weight loss; and 4, > 15% weight loss, liquid stool, and/or obvious blood in the stool. The daily DAI score was calculated by taking the arithmetic mean of three variables in each group (Fouad et al. 2021). Occult blood in stools was detected by the guaiac method using the fecal occult blood test (Guimarães et al. 2019).

### 2.4 Colon Mass Index Measurement

Colon mass index was calculated as the ratio of colon weight to body weight recorded on the last day (Soliman et al. 2018; Gao et al. 2021). Results were expressed as milligram tissue weight per gram animal weight (mg/g).

### 2.5 Colon Macroscopic Evaluation

Macroscopic lesions in the distal 8 cm of the colon samples were examined and scored according to areas of hyperemia, ulceration, and inflammation (Karakoyun et al. 2011; Kolgazi et al. 2013).

### 2.6 Colon Microscopic Evaluation

To determine damage at the cellular level, the colon tissues of the rats were histopathologically evaluated. Tissues were fixed in 10% neutral buffered formaldehyde. Routine tissue processing was performed on a tissue processor. Subsequently, the tissues were embedded in paraffin blocks, and 5‐μm‐thick sections were obtained from paraffin blocks on slides using a rotary microtome. The slides were brought to distilled water and stained with hematoxylin and eosin (H&E), Masson's trichrome (BO 04‐010802, Bio‐Optica, Italy), and periodic acid–Schiff (PAS) (BO 04‐130802, Bio‐Optica, Italy). Colon tissues were examined for ulcers, crypt damage, congestion, edema, and infiltration. Evaluations were performed semiquantitatively under a light microscope, and findings were scored histologically (Karakoyun et al. 2011; Kolgazi et al. 2013). At least four microscopic fields were examined for scoring each sample, and the maximum score for all samples was 14. Photographs of the examined tissues were taken at different magnifications.

### 2.7 Colon IHC Zonula Occludens (ZO)‐1 Assessment

All 5‐μm‐thick sections obtained from paraffin blocks to positively charged slides for IHC were deparaffinized and rehydrated by passing through xylene and a descending alcohol series after being kept in an oven at 60°C overnight. Tris buffer saline (TBS), which is used as a wash buffer and for antigen retrieval, was performed using a microwave oven at 800 W for 2 min 10 times in a Tris–EDTA buffer solution. Endogenous peroxidase activity was suppressed using hydrogen peroxide block. After washing with TBS, the slides were incubated with an Ultra V Block solution to prevent nonspecific background staining. Subsequently, the sections were incubated with the primary antibody (ab221547, Abcam, UK) at a 1:400 dilution overnight at 4°C. The slides were taken to 23°C and washed with TBS four times. The secondary antibody (TP‐125‐HL, Biotinylated Goat Anti‐Polyvalent, USA) was added to the sections, incubated for 10 min, and washed with TBS. Streptavidin peroxidase was added, incubated for 10 min, and washed with TBS. Color formation was provided using the DAB Chromogen Kit (TA‐125‐HS, Thermo Fisher, USA), washed in distilled water, and subsequently counterstained with hematoxylin. For evaluation, four random areas at ×400 magnification were determined in each section and examined under a light microscope for semiquantitative evaluation. The percentage of stains on colon glandular epithelial cells in each area of the preparations was calculated. For each area, staining intensity was determined as no staining (0), weak staining (+), moderate staining (++), and strong staining (+++), and the sections were evaluated according to their immune positivity using the calculated H score (intensity × percentage).

### 2.8 Colon Myeloperoxidase Assay

Colon myeloperoxidase (MPO) assay was based on the reduction of hydrogen peroxide oxidized by MPO with O‐dianisidine hydrochloride and measurement of the absorbance of this reduced product at 460 nm (Pazar et al. 2016). The resulting supernatants were measured using a spectrophotometer at 460 nm, and results were expressed as units per gram tissue (U/g).

### 2.9 Statistical Analysis

Values were presented as means ± SD. Data were analyzed using SPSS (IBM SPSS Statistics, version: 25). For descriptive analysis, the arithmetic means and standard deviations of the groups were calculated. One‐way analysis of variance and Tukey's tests were used when the distributions were parametric. The Kruskal–Wallis and Mann–Whitney *U*‐tests were used for nonparametric distributions. *p* values of < 0.05 were considered statistically significant.

## 3 Results

### 3.1 DAI

**FIGURE 2:**
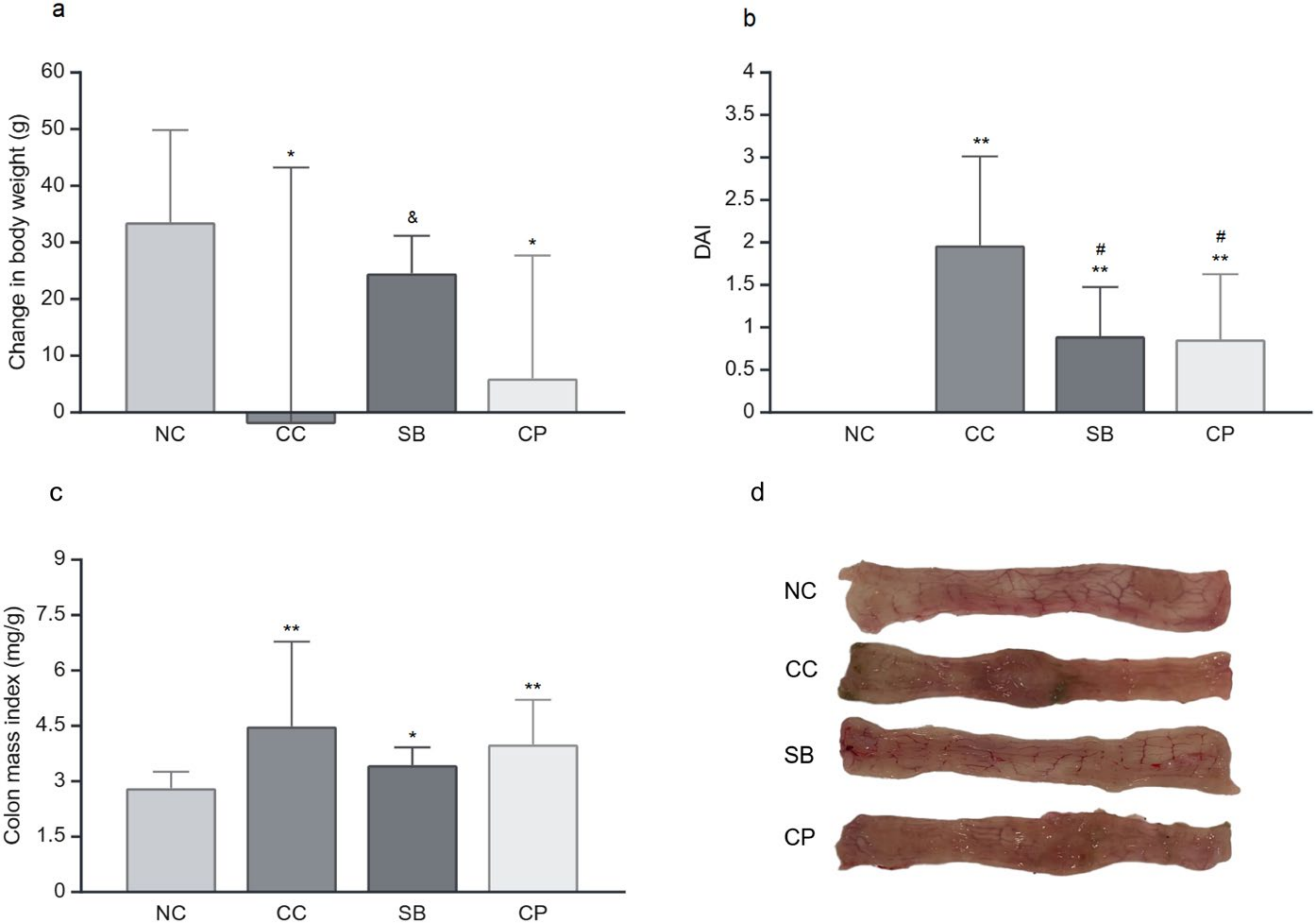
Effects of treatments on (a) changes in body weight, (b) disease activity index, (c) colon mass index, and (d) macroscopic appearance of distal 8 cm colons in colitis rats. CC, colitis control; CP, collagen peptidesNC, normal colitis; SB, *S. boulardii*. Data are presented as mean ± SD with *p < 0.05 and **p < 0.01 compared to the NC group, #*p* < 0.05 and ##*p* < 0.01 compared to the CC group, and *p* < 0.05 compared to the CP group.

Adult and healthy rats were expected to gradually gain weight. Consistently, the weights of the rats in the NC group continued to increase over the course of the experiment (33.44 ± 16.25). After 7 days, the weight changes of the rats in the CC group were significantly decreased owing to severe inflammation (−2.25 ± 45.33; *p <* 0.05). The SB treatment increased the changes in weight by reducing weight loss of rats (24.44 ± 6.60 g), ensuring no significant difference to the NC. Similar to SB, CP treatment also had an effect on preventing weight loss (5.90 ± 21.67 g). On the other hand, the SB treatment was significantly more effective than CP in preventing weight loss (*p <* 0.05; Figure 2a).

In the CC group, stool consistency score (3.00 ± 1.51; *p <* 0.01) and blood in the stool score increased (1.75 ± 0.70; *p <* 0.05). SB and CP treatments significantly improved stool consistency (1.11 ± 1.45; *p < 0.05* and 0.44 ± 1.33; *p <* 0.01) and slightly improved blood in the stool (1.55 ± 0.88; 1.55 ± 0.88).

To determine disease severity, DAI was calculated separately for each day. During the experiment, a significant difference was observed between the NC group and all other groups in terms of DAI. On the seventh day, the disease severity of the CP (0.85 ± 0.76; *p <* 0.05) and SB (0.88 ± 0.57; *p <* 0.05) groups significantly decreased compared with that of the CC group (1.95 ± 1.04). No difference was noted between the treatment groups (Figure 2b).

### 3.2 Colon Mass Index Assessment

To assess the degree of edema and severity of inflammation of the colon, the colon mass index was calculated (Soliman et al. 2018). Compared with the NC group (2.79 ± 0.44 mg/g), the index was substantially increased in the CC group (4.47 ± 2.28 mg/g; *p <* 0.01). Compared with the CC group, CP and SB treatments alleviated edema in the colon, and the colon mass index improved (3.98 ± 1.20 mg/g and 3.42 ± 0.47 mg/g, respectively). Compared with the CP group, the effect of the SB treatment on the colon mass index was slightly greater (Figure 2c).

### 3.3 Colon Macroscopic Evaluation

**FIGURE 3:**
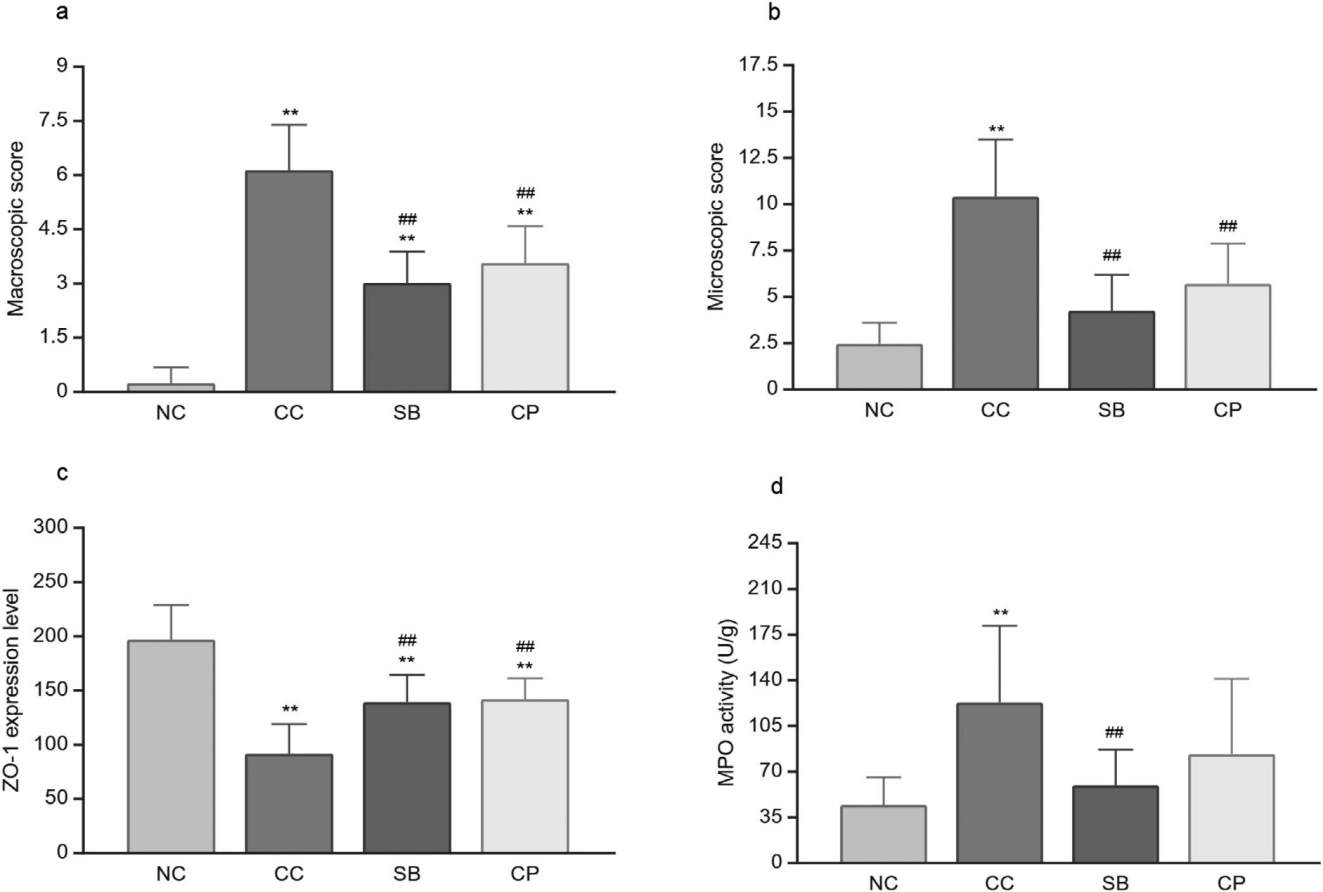
Effects of treatments on colon damage in AA‐induced colitis in rats. (a) Colon macroscopic score, (b) microscopic score, (c) ZO‐1 expression level, and (d) MPO activity. NC, normal colitis; CC, colitis control; CP, collagen peptides; SB, *S. boulardii*; MPO, myeloperoxidase; ZO‐1, zonula occludens‐1. Data are presented as mean ± SD with **p < 0.01 compared to the NC group, and, ## *p* < 0.01 compared to the CC group.

The CC group (6.12 ± 1.24) showed a significantly increased colon macroscopic damage compared with the NC group (0.22 ± 0.44; *p <* 0.01). However, CP and SB treatments significantly reduced colon hyperemia, ulceration, and inflammation, thereby resulting in marked tissue healing (3.55 ± 1.01 and 3.00 ± 0.86, respectively; *p <* 0.01). Regarding macroscopic improvement, no significant difference was observed between SB and CP treatments (Figures 2d and 3a).

### 3.4 Colon Histologic Evaluation

**FIGURE 4:**
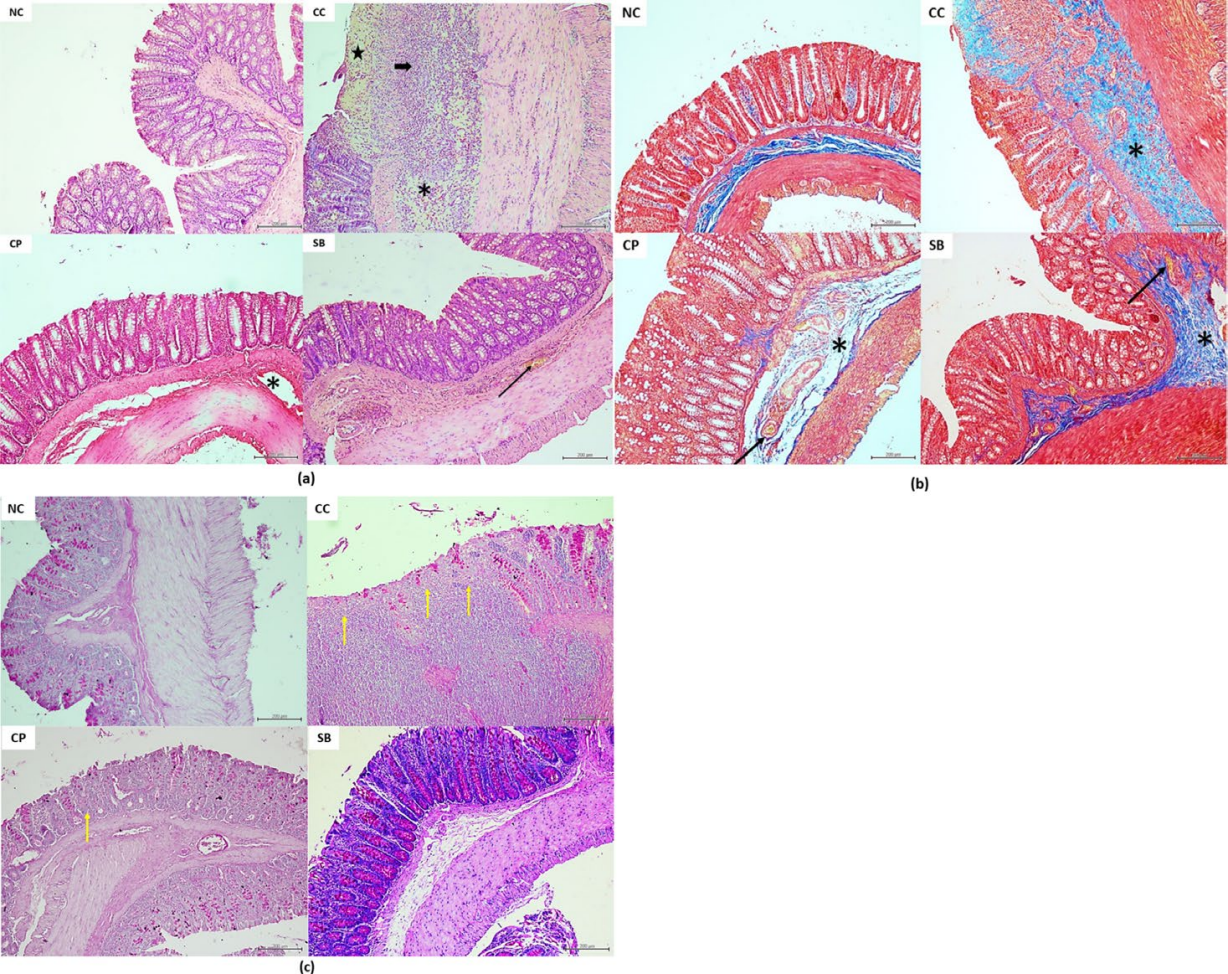
Light microscopic examination of the colon mucosa in the experimental groups. (a) H&E stain. (b) Masson's trichrome stain. (c) PAS stain. The NC group showing a regular morphology of the colon mucosa. The CC group shows extensive ulcer (star), transmural inflammatory cell infiltrates (thick arrow), severe submucosal edema (asterisks), and cyrpt damage with loss of goblet cells (yellow arrow). The CP and SB groups show mild‐to‐moderate changes, including vascular congestion (thin arrow) and mild edema (asterisk), similar to the CC group. All bars are 200 μm. CC, colitis control; CP, collagen peptides; H&E, hematoxylin and eosin; NC, normal colitis; PAS, periodic acid–Schiff; SB, *S. boulardii*.

H&E‐, PAS‐, and Masson's trichrome‐stained samples were used for histologic evaluations. The CC group (10.37 ± 3.06) showed significantly higher colon microscopic damage scores than the NC group (3.57 ± 3.45; *p <* 0.01). Masson's trichrome staining revealed increased submucosal edema in the CC group. Increased inflammatory cell infiltrates were observed in the H&E‐stained samples of the CC group, and we noted increased ulcer and crypt damage in the CC group with PAS staining. The CC group more frequently showed particularly extensive ulcers and entire crypt damage than the CP and SB groups. CP and SB treatments showed histologically significant improvement in colitis (5.66 ± 2.17 and 4.22 ± 1.92, respectively; *p <* 0.01). However, no significant difference was noted between the NC and the CP and SB groups (Figures 3b and 4).

### 3.5 Colon IHC ZO‐1 Assessment

**FIGURE 5:**
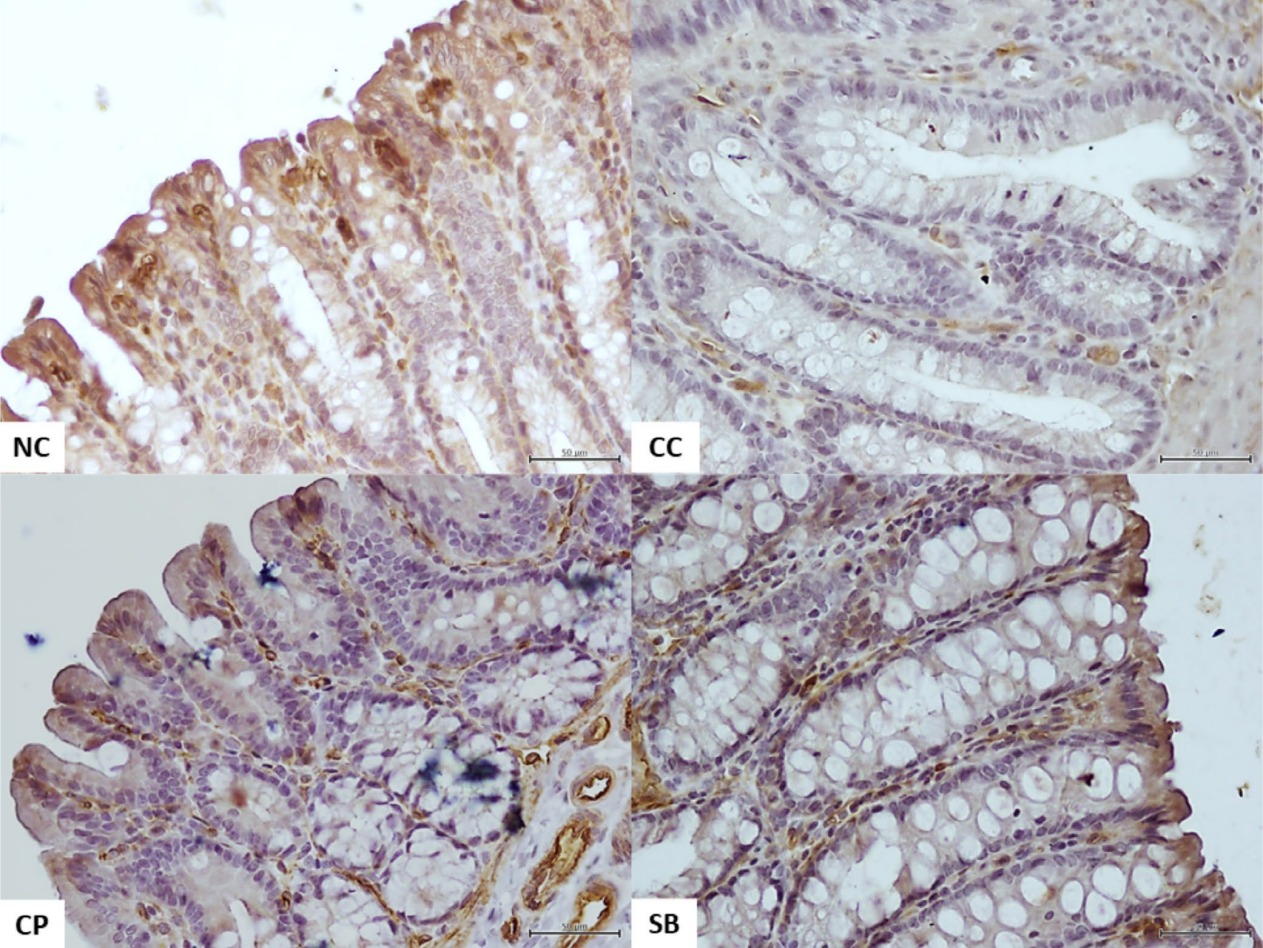
ZO‐1 immunohistochemical staining image of the representative colon sections from the experimental groups. The NC group showing a strong expression of ZO‐1 in the crypt epithelium. The CC group shows a very weak expression, and the CP and SB groups show a moderate expression of ZO‐1 in the crypt epithelium. All bars are 50 μm. CC, colitis control; CP, collagen peptides; NC, normal colitis; SB, *S. boulardii*; ZO‐1, zonula occludens.

To assess between‐group differences in colon epithelial ZO‐1 protein expression, IHC analysis was performed. The levels were markedly lower in all colitis groups than those in the NC group (196.42 ± 31.93; *p <* 0.01). However, the CP and SB groups (141.48 ± 19.02 and 138.70 ± 25.08, respectively; *p <* 0.01) showed significantly increased colon ZO‐1 protein levels compared with the CC group (91.24 ± 27.14), indicating that the treatments significantly restored the colon barrier function. The CP treatment was more effective than SB in re‐expressing the ZO‐1 protein; however, no statistically significant difference was observed between them (Figures 3c and 5).

### 3.6 Colon MPO Assay

The MPO assay is frequently used as an indicator of inflammation in colon samples. Colon MPO levels significantly increased in the CC group (122.50 ± 58.65 U/g) compared to the NC group (43.90 ± 21.26; *p <* 0.01). SB and CP treatments improved MPO levels by reducing neutrophil infiltration in the colon (59.04 ± 27.40 U/g and 83.02 ± 57.60 U/g, respectively). Compared with the CC group, only the change in the SB group was significant (*p <* 0.01). SB and CP treatments elicited similar MPO levels to the healthy controls (Figure 3d).

## 4 Discussion

UC is a chronic inflammatory condition characterized by colon inflammation and ulcer formation. It triggers a localized and intense immune response, leading to macroscopic lesions such as edema and ulceration (Matsuoka et al. 2018). The inflammatory process in UC is driven by elevated levels of proinflammatory cytokines (Ansari et al. 2021). Additionally, oxidative stress plays a key role by increasing the production of reactive oxygen species and their metabolites, further exacerbating inflammation. Oxidative stress increases neutrophil infiltration, which causes mucosal edema, mucosal damage (Balmus et al. 2016), and, eventually, significant colon inflammation (Rehman et al. 2022).

To date, no treatment has been discovered that eliminates UC. Therefore, research continues to explore new pharmacological and nutritional therapies to improve disease management (Kotlyar et al. 2011). Animal disease models allow for the preclinical identification of the efficacy and safety profiles of new treatment options. The AA‐induced colitis model is widely used (Owusu et al. 2020). Intrarectal administration of AA impairs the colon's ability to absorb liquid from stool, leading to a large volume of watery stool. AA induces oxidative damage and tissue destruction in the colon mucosa, triggering a cascade of inflammatory responses. It causes tissue necrosis, erosion, severe ulceration, mucosal atrophy, and inflammatory cell infiltration, thereby initiating the inflammatory cascade (Rafeeq et al. 2021). Additionally, AA disrupts intestinal barrier function by modulating the mucosal immune system (Bastaki et al. 2021). The histopathological changes in AA colitis are similar to those in human UC (Oluwagbamila et al. 2023). It also includes typical clinical symptoms such as severe diarrhea, bloody stools, and weight loss (Shahid et al. 2022). This study investigated the effects of collagen peptides and *S. boulardii* CNCM I‐745 on disease progression in an AA‐induced colitis model.

The study confirmed the induction of an acute colitis model by observing clinical symptoms in rats administered AA intrarectally, including weight loss, blood in the stool, and altered stool consistency. DAI is commonly used to assess the severity of AA‐induced colitis (Ansari et al. 2021). Additionally, microscopic examination of colon tissue revealed inflammatory cell infiltration and tissue damage (Owusu et al. 2020). Consistent with the findings of Mohamed et al. (2022), our study identified histopathological changes such as severe colon ulceration, crypt damage, neutrophil and leukocyte infiltration, goblet cell loss, and elevated DAI levels.

Body and colon weight changes, bloody stools, and diarrhea caused by AA are believed to be sensitive indicators of the degree and severity of inflammation (Bastaki et al. 2021). In healthy adult rats, it is normal for their body weight to increase day after day. Bloody stool and diarrhea in colitis were previously thought to cause fluid loss and thus reduce body weight (Ansari et al. 2021). Indeed, findings in this direction have emerged in colitis rats. The study found that blood in stool and diarrhea increased significantly with a decrease in this weight change in colitis rats, which is consistent with previous studies (Owusu et al. 2020; Otu‐Boakye et al. 2023). Body weight loss in rats is also associated with histopathological changes in colitis (Gonzalez‐Rey et al. 2006). Body weight change increased positively in both treatment groups. Furthermore, diarrhea and blood in the stool were found to be reduced, with a statistically significant decrease in stool consistency. Decreasing blood in the stool may indicate a decrease in mucosal ulceration and healing (Ramadass et al. 2016).

Increased colon weight in UC indicates inflammation, edema, and wall thickening (Abiodun and Oshinloye 2017). According to Rodríguez‐Nogales et al. (2018), the colon mass index correlates with the severity of colon damage. The results showed that AA‐induced colitis rats had a significantly higher DAI score and colon mass index. Gao et al. (2021) found a similar result in DSS colitis mice. In our study, the oral administration of both treatments resulted in a significant decrease in DAI and colon mass index values in colitis rats. Mucosal healing has been identified as a significant and reliable predictor of treatment efficacy and long‐term outcomes in IBD (Rutgeerts et al. 2007). As a result, the colon was histologically examined in each treatment group. The treatments were found to be effective in reversing colon mucosal damage. As a result, it was demonstrated that they maintained the structural architecture and function of the colon mucosal epithelium. These results may be attributed to collagen peptides and *S. boulardii* treatments that reduce inflammatory mediators and oxidative stress.

Our study corroborated previous findings that AA induces macroscopic changes in the colon, including wall thickening, hyperemia, ulceration, edema, and necrosis (Ahmed et al. 2022; Owusu et al. 2020). Macroscopic examination of the treated groups revealed a significant reduction in hyperemia, ulceration, and inflammatory lesions, which was consistent with histopathological findings. Similarly, Rahabi et al. (2022) and Zhu et al. (2018) found that collagen peptide treatment significantly reduced macroscopic and histological damage in DSS colitis. *S. boulardii* treatment has been shown to improve DSS colitis (Zhou et al. 2018; Rodríguez‐Nogales et al. 2018) and TNBS colitis (Lee et al. 2009) at both macroscopic and histopathological levels.

In UC, a large number of neutrophils infiltrate the inflamed mucosa and accumulate in the epithelium, causing mucosal damage. It disrupts the epithelial barrier, resulting in the production of inflammatory mediators such as MPO (Zhou and Liu 2017). MPO levels were found to be elevated in the colon mucosa of UC patients (Turkay and Kasapoglu 2010). MPO is a commonly found enzyme in neutrophils. It produces hypochlorous acid, a potent oxidant that causes oxidative stress, inflammation, and tissue damage (Ndrepepa 2019). Increased MPO levels were also linked to DAI scores in the DSS‐induced colitis model (Chen, Hou, et al. 2017; Chen, Zhou, et al. 2017). In addition to DAI scores, our study found a significant increase in MPO activity in the colitis group, which is an indicator of inflammation.

Colon MPO activity decreased in both treatment groups. As MPO is a well‐established marker of colon inflammation and injury and is closely associated with neutrophil infiltration (Krawisz et al. 1984; Mullane et al. 1985), this reduction suggests that the tested agents exert anti‐inflammatory effects (Liu and Wang 2011). Notably, *S. boulardii* treatment significantly improved MPO levels, further supporting the histological findings of reduced infiltration of inflammatory cells. Previous studies have also demonstrated that collagen peptides (Azuma et al. 2014; Zhu et al. 2018) and *S. boulardii* (Rodríguez‐Nogales et al. 2018; Gao et al. 2021) lower tissue MPO levels in rodent models of colitis. Our findings indicate that these treatments modulate immune responses through anti‐inflammatory mechanisms, highlighting their potential as therapeutic agents for UC.

Previous research has shown that AA‐induced colitis reduces colon permeability and increases bacterial translocation into the colon by causing epithelial damage and reducing goblet cells (Fournier and Parkos 2012; Fawzy et al. 2013). Depletion of goblet cells is thought to be the hallmark of UC because it causes mucosal barrier dysfunction (Nowarski et al. 2015). In the present study, colon sections from AA colitis rats showed a decrease in goblet cells. It was found that goblet cells were preserved in the mucosal epithelium of rats in the treatment groups. This finding was interpreted as yet another clue to the role of treatments in the management of UC. Collagen peptides and *S. boulardii* treatments are believed to help protect the mucosal epithelium by promoting goblet cells (Rehman et al. 2022). These aspects are important for therapeutic purposes because epithelial damage and barrier permeability in goblet cells have been linked to inflammation (Jang et al. 2022).

Colon ZO‐1 density decreased in AA colitis rats, indicating colon barrier disruption (Owusu et al. 2020; Soliman et al. 2018). Tight junction proteins are structures found between colon epithelial cells that are essential for intestinal barrier function (Li et al. 2022). Tight junctions are primarily composed of transmembrane proteins such as occludin, claudins, junctional adhesion molecules, and ZO‐1 (Faghih et al. 2024). Impaired tight junction barrier function has been linked to the pathogenesis of IBD, resulting in increased epithelial permeability, bacterial translocation, subsequent leukocyte accumulation, cytokine release, and inflammation (Martini et al. 2017). Previous research has shown that patients with IBD have lower levels of important tight junction proteins such as occludin and ZO‐1 and higher levels of intestinal permeability (Tan et al. 2019).

In this study, treatment with bovine collagen peptide and *S. boulardii* significantly reversed the decrease in colon ZO‐1 levels in AA colitis, reducing colon permeability and helping to restore intestinal epithelial architecture. This finding is consistent with previous studies, except Li et al. (2022), which found the opposite. Guo et al. (2023) found that treating mice with DSS‐induced colitis with oral cod skin collagen peptide improved ZO‐1 and occludin expressions in the colon mucosa. Chen et al. (2019) demonstrated that pollock skin‐derived collagen peptides improved burn‐induced disruptions in ZO‐1 and occludin expression and localization while supporting intestinal barrier integrity. Probiotic treatments are known to promote intestinal homeostasis by suppressing pathogenic bacteria, strengthening the epithelial barrier, and regulating inflammatory cytokines production (Liang et al. 2014). Previous reports have stated that *S. boulardii* treatment enhances ZO‐1 expression levels and IHC distributions in rodents with DSS colitis (Rodríguez‐Nogales et al. 2018; Zhou et al. 2018; Dong et al. 2019; Gao et al. 2021). Collagen peptides and *S. boulardii* may have therapeutic potential for UC by improving mucosal repair and strengthening the intestinal barrier.

The study's limitations included a lack of direct examination of colon microbiota and proinflammatory cytokine levels. Previous research has found higher levels of proinflammatory cytokines in DSS colitis and UC patients (Biesiada et al. 2012; Zhang et al. 2017). TNF‐α and IL‐1β cytokines can cause intestinal inflammation by increasing permeability and disrupting the tight junction protein barrier (Lee 2015). Therefore, targeting the reduction of proinflammatory cytokine levels is a rational approach to the treatment of UC (Moschen et al. 2019). Furthermore, IBD has been linked to reduced colon microbiota diversity and composition (Van Averbeke et al. 2022). However, *S. boulardii* treatment has been shown to restore the structural and compositional changes in intestinal microflora induced by DSS (Xu et al. 2023). Fish collagen peptides have been shown to suppress the abnormal expression of colon tight junction proteins in DSS colitis while improving intestinal microbiota dysbiosis (Yang et al. 2024). Given these significant findings, we intend to investigate colon proinflammatory cytokine levels and microbiota in AA colitis. Therefore, the molecular mechanisms underlying the effects of collagen peptides and *S. boulardii* CNCMI‐745 treatments on immune modulation, tight junction protein regulation, and intestinal microbiota interactions warrant further investigation.

## 5 Conclusion

This is the first study to reveal the effects of dietary types 1 and 3 bovine collagen peptide and *S. boulardii* CNCMI‐745 treatments in AA‐colitis rats. Findings of our study have indicated that collagen peptide and *S. boulardii* CNCM‐745 has a beneficial effect on the course of colitis in rats by reducing disease activity, colon inflammation, and colon permeability. Collagen peptide and *S. boulardii* are potential therapeutic candidates for UC treatment. Future studies should investigate the long‐term efficacy and clinical applicability of collagen peptides and *S. boulardii* at different doses.

## Author Contributions

**Öykü Altınok:** conceptualization (lead), data curation (lead), formal analysis (lead), investigation (lead), methodology (lead), resources (lead), validation (lead), writing – original draft (lead), writing – review and editing (lead). **Murat Baş:** funding acquisition (lead), resources (supporting), supervision (lead), validation (supporting), writing – original draft (supporting). **Elif Gelenli Dolanbay:** investigation (supporting), methodology (supporting), visualization (supporting), writing – review and editing (supporting). **Meltem Kolgazi:** investigation (supporting), methodology (supporting), visualization (supporting). **Tugay Mert:** investigation (supporting), methodology (supporting). **Ünal Uslu:** methodology (supporting).

## Ethics Statement

This study protocol was reviewed and approved by Yeditepe University Animal Experiments Local Ethics Committee, approval number 2021/08‐10.

## Conflicts of Interest

The authors declare no conflicts of interest.

## Data Availability

The data that support the findings of this study are available from the corresponding author upon reasonable request.
